# Insight into regulation of adventitious root formation by arbuscular mycorrhizal fungus and exogenous auxin in tea plant (Camellia sinensis L.) cuttings

**DOI:** 10.3389/fpls.2023.1258410

**Published:** 2023-09-18

**Authors:** Weili Chen, Wenshu Shan, Tingting Niu, Tao Ye, Qinyu Sun, Jiaxia Zhang

**Affiliations:** Tea Research Institute, Anhui Academy of Agricultural Sciences, Huangshan, China

**Keywords:** arbuscular mycorrhizal fungus, tea plant, adventitious root, auxin transport, auxin signal transduction

## Abstract

**Introduction:**

Adventitious root (AR) development, affected by various biotic and abiotic factors, is the most important procedure in tea plant (*Camellia sinensis* L.) cutting propagation. Establishing symbiotic relationships with most terrestrial plants, AMF (Arbuscular mycorrhizal fungus) can mediate the AR formation of several herbaceous and woody plants in previous studies.

**Methods:**

In this paper, effects of combined application of AMF and exogenous auxin on AR formation of cuttings from different tea plant varieties (‘Pingyangtezao’, ‘Longjing 43’ and ‘Longjingchangye’) were studied. Then we also performed RNA-Seq analysis with ‘Pingyangtezao’ cuttings aiming to find the possible auxin-related pathway of AM fungal regulation on AR formation. To accurately uncover the regulatory mechanism of AMF on AR formation of tea cuttings, rooting process were separated into four stages (S0, non-rooting; S1, AR protrusion; S2, AR formation and S3, AR elongation) at the same sampling time.

**Results and Discussion:**

Results showed that IBA treatment increased the mycorrhizal colonization rate, especially in ‘Pingyangtezao’ variety (from 37.58% to 46.29%). Both inoculating AMF and addition of IBA promoted the AR formation, and rooting of different tea plant varieties showed different dependence on auxin. AMF could alleviate the effect of auxin-related inhibitors (2,3,5-triiodobenzoic acid, L-α-(Aminooxy)-β-phenylpropionic acid and α-(phenylethyl-2-oxo)-IAA) on rooting of tea cuttings, even though the colonization of AMF was hindered at various degrees. Transcriptomic analysis showed that different numbers of differentially expressed genes (DEGs) at various rooting stages of tea cuttings with the most at S2 stage (1360 DEGs), indicating the increasing regulation by AMF with the development of AR. Similar trend was found in auxin-related DEGs, and family genes of *YUC*, *GH*, *PIN*, *LAX*, *SAUR*, *AUX*, and *ABP* involved in the AM fungal regulation on AR formation of tea cuttings. Additionally, AMF strongly mediated auxin transport and signal transduction pathways in tea cuttings as showed by the results of correlation analysis. Overall, interaction of AMF and exogenous auxin in promoting rooting and the preliminary mechanism of AMF regulating AR formation of tea cuttings was deciphered in this paper, which may provide a basis for further deep mechanistic research and cutting propagation of tea production.

## Introduction

Adventitious root (AR), appeared from non-root tissues, such as stems or leaves, plays a crucial role in the growth and development of woody plants by serving various important functions. One significant importance of AR is the role in vegetative propagation, which can enable the establishment of a new individual identical to the parent plant to keep the same excellent characteristics. AR formation involves a complex interplay of hormonal and environmental factors. The process begins with the activation of dormant cells in the stem or leaf tissue, triggered by specific plant hormones, particularly auxin. Auxin promotes cell division, elongation, and differentiation, leading to the formation of root primordia. These primordia later develop into fully functional adventitious roots. Genes of auxin biosynthesis and metabolism (flavin-containing monooxygenase *YUC* and acyl acid amido synthetase *GH3*, transport (PIN-FORMED protein *PIN* and PIN-likes *PILS*) and signal transduction (auxin early responsive protein *AUX/IAA*, auxin response factor *ARF*, and auxin-responsive protein *SAUR*) are all closely correlated with the adventitious root formation as showed in many previous studies (Yin et al., 2020; Hu et al., 2023; Shen et al., 2023; Wei et al., 2023; Zhu et al., 2023). Moreover, auxin also interacts with other plant hormones such as cytokinin, ethylene and jasmonic acid to jointly regulate the formation of AR. Recently, an extensive and distinctive book on the environmental, physiological and chemical factors associated with adventitious root formation in cuttings was provided, and partial mechanisms about AR formation in woody plants was also reviewed (Husen, 2022).

Originated in the southwest of China, tea has proliferated to over 100 countries worldwide (Zhang et al., 2023). Tea plants (*Camellia sinensis* L.) generally rely on the vegetative propagation to produce seedlings for widely cultivating because of self-incompatibility and separation of individual progeny traits (Wang et al., 2022a). Therefore, adventitious root formation is of great importance in the spread of new and superior varieties. Various tea plant varieties exhibit different abilities in AR formation (Duan et al., 2017; Duan et al., 2018), which are closely associated with plant hormones, enzymes, nutrients, and the positioning of AR primordium in cuttings (Ma et al., 2015; Miao et al., 2022). Depending on the time of formation, AR primordia were divided into two types: latent root primordia and induced root primordia, and the latter type is often found in woody plants which show difficulty in rooting, such as *Ginkgo biloba* and *Paeonia suffruticosa* ‘Taipinghong’ (Miao et al., 2022). Additionally, according to the position of AR primordia, callus rooting, cortex rooting and comprehensive rooting are the main three types in woody plants (Wang et al., 2022a; Miao et al., 2022), such as poplar. The most prevalent rooting type found in tea plants is the comprehensive rooting type, and the level of difficulty in rooting is typically associated with the proportions of callus rooting and cortex rooting types, which may cause the difference in rooting ability of various varieties. Nowadays, many studies have focused on the mechanisms of AR formation in tea plants, and preliminary results all showed that auxin is a key factor involved in this process (Wang et al., 2022b; Fu et al., 2023; Hu et al., 2023).

Arbuscular mycorrhizal fungus (AMF), forming symbiosis with almost 80% of terrestrial plants, can exert both directly and indirectly growth promotion effect in normal or abnormal conditions and elevate the resistance to diseases and pests (Du et al., 2022; Eck et al., 2022). In our previous research, we found that inoculating with AMF significantly increased the resistance to anthracnose in tea seedlings through the regulation of plant hormones and the antioxidant system (especially peroxidase) (Chen et al., 2023). Because tea plants are mycorrhizal-dependent plants (Liu et al., 2023a), AMF plays a great role in many aspects. So far, a few researchers have studied the effect of AMF on the rooting of tea plant cuttings (Liu et al., 2007; Gao et al., 2023). Gao et al. (2023) found that inoculation with AMF individually or hormones treatment alone significantly enhance mycorrhizal colonization, growth and physiobiochemical characteristics of tea cutting seedlings, including the formation of AR. However, the underlying mechanism of AM fungal regulation on AR formation was not well discussed yet. Therefore, in this paper, we aimed to uncover the preliminary mechanism of AMF regulating rooting ability of tea plant cuttings by centering on auxin, which is the most important phytohormone in the development of AR formation according to previous studies (Hu et al., 2023; Shen et al., 2023). Moreover, we firstly separated the process of AR development into 4 stages of non-rooting, AR protrusion, AR formation and AR elongation (which can be seen in part of Materials and methods) at the same sampling time in order to clarify the underlying mechanism. Different auxin inhibitors, such as 2,3,5-triiodobenzoic acid (TIBA) (Xu et al., 2016), L-α-(Aminooxy)-β-phenylpropionic acid (AOPP) (Higashide et al., 2014; Yang et al., 2021) and α-(phenylethyl-2-oxo)-IAA (PEO-IAA) (Hayashi et al., 2008), were also used for elucidating the role of auxin in AM fungal regulation on AR formation. Among these, TIBA interferes with auxin transport (Sussman and Goldsmith, 1981), while AOPP inhibits auxin biosynthesis, which is known as an inhibitor of phenylalanine ammonia-lyase (Higashide et al., 2014). PEO-IAA, an auxin antagonist, can bind to transport inhibitor response 1/auxin signaling F-box proteins (TIR1/AFBs) (Nishimura et al., 2009). All results in our paper will provide a theoretical reference for the propagation of tea cuttings in future and lay a solid foundation for the production of mycorrhizalized tea seedlings.

## Materials and methods

### Experimental materials

*Rhizophagus intraradices* BGC JX04B, used as the AMF isolate, was provided by the Beijing Academy of Agriculture and Forestry Sciences (Chen et al., 2021). Consisted of a mixture of spores, mycelium, fine root segments and growth medium, the inoculum was prepared by propagating the isolate with sorghum (*Sorghum bicolor* L. Moench) as a host for 2 months in the greenhouse.

In this paper, we conducted two experiments in total. Tea plant varieties of ‘Pingyangtezao’, ‘Longjing 43’ and ‘Longjingchangye’ were used as the plant materials in the first experiment in order to observe the effect of AMF and auxin on rooting of cuttings from different tea plants. In the second experiment, tea plant variety ‘Pingyangtezao’ with easy-rooting feature was used as the plant material according to the results of the first experiment to clarify the role of auxin involved in the regulation of AMF on adventitious root formation of tea plant cuttings. Semi-woody shoots of the chosen tea plant varieties with approximately 7-year-old under identical growth and management conditions, which are usually reddish brown, were collected from the tea plant germplasm resource nursery of the Tea Research Institute, Anhui Academy of Agricultural Sciences, Huangshan, China (29°41’18”E, 118°15’39”N).

### Experimental design

In the laboratory, each shoot was segmented into single node cuttings with approximately 3.5 cm in length. In the first experiment, 300-mL containers were filled with autoclaved (121°C, 2 h) river sand, and nutrient solutions for cutting experiment were as follows: 40 mg·L^-1^ N (NH_4_)_2_SO_4_, 0.31 mmol·L^-1^ P (KH_2_PO_4_), 40 mg·L^-1^ K (KH_2_PO_4_ and K_2_SO_4_) according to Pan and Konish (1995) with minor alterations. pH was adjusted to 5.8 with HCl. After moistening the cutting medium with nutrient solutions, 6-8 single-node cuttings were inserted into each container. For the second experiment, 300-mL containers were replaced by plant culture box with lid (length * width * height = 120 * 120 *120 mm), and about 400 mL autoclaved river sand was added into each box.

For mycorrhizal treatment, 5 g AMF inoculum or sterilized inoculum was added into the river sand in the middle of the container to generate mycorrhizal or non-mycorrhizal seedlings, respectively. To avoid the potential influence of other microbes, we also added 5 mL AM fungal inoculum filtrates (25-mm filter) to the non-mycorrhizal treatment. Moreover, two clover (*Trifolium repense* L.) seedlings (3-5 clover seedlings in the second experiment), as pre-hosts of AMF, were planted in the center of each container or plant culture box.

For auxin treatments, 20 mg·kg^-1^ indole-3-butyric acid (IBA) (Xu et al., 2016) was used as the external auxin. 5 mg·kg^-1^ 2,3,5-triiodobenzoic acid (TIBA) (Xu et al., 2016), 20 µM L-α-(Aminooxy)-β-phenylpropionic acid (AOPP) (Higashide et al., 2014; Yang et al., 2021), 10 μM α-(phenylethyl-2-oxo)-IAA (PEO-IAA) (Hayashi et al., 2008) were used as the inhibitors of auxin transport, biosynthesis and signal transduction, respectively, with minor alterations.

All treatments in this paper were listed in **Table 1**. Specifically, in the first experiment, 10 treatments with 4 containers in each were generated: C (non-mycorrhizal treatment), T (mycorrhizal treatment), C+IBA, C+TIBA, C+AOPP, C+PEO-IAA, T+IBA, T+TIBA, T+AOPP, and T+PEO-IAA. While in the second experiment, treatments of C and T were arranged with using tea plant variety ‘Pingyangtezao’, wherein each treatment was composed of 6 plant culture boxes. All containers were first distributed in nursery boxes (54 cm length × 28 cm width × 24 cm height) with cap to keep high humidity, and then placed in an artificial climate culture chamber with a light intensity of 5,000 lx and photoperiod of light:dark = 16:8 h. The temperature and relative humidity in the chamber were 28°C and 80%, respectively. For the second experiment, all plant culture boxes were directly placed into the artificial climate culture chamber.

**Table 1:** The design of the two experiments. IBA, TIBA, AOPP and PEO-IAA indicated indole-3-butyric acid, 2,3,5-triiodobenzoic acid, L-α-(Aminooxy)-β-phenylpropionic acid, and α-(phenylethyl-2-oxo)-IAA, respectively.

Different experiments	Treatment	Description
First experiment	C	non-mycorrhizal treatment
	T	mycorrhizal treatment
	C+IBA	non-mycorrhizal treatment with adding IBA
	C+TIBA	non-mycorrhizal treatment with adding TIBA
	C+AOPP	non-mycorrhizal treatment with adding AOPP
	C+PEO-IAA	non-mycorrhizal treatment with adding PEO-IAA
	T+IBA	mycorrhizal treatment with adding IBA
	T+TIBA	mycorrhizal treatment with adding TIBA
	T+AOPP	mycorrhizal treatment with adding AOPP
	T+PEO-IAA	mycorrhizal treatment with adding PEO-IAA
Second experiment	C	non-mycorrhizal treatment
	T	mycorrhizal treatment

### Sampling and morphological indexes determination

**Figure 1:**
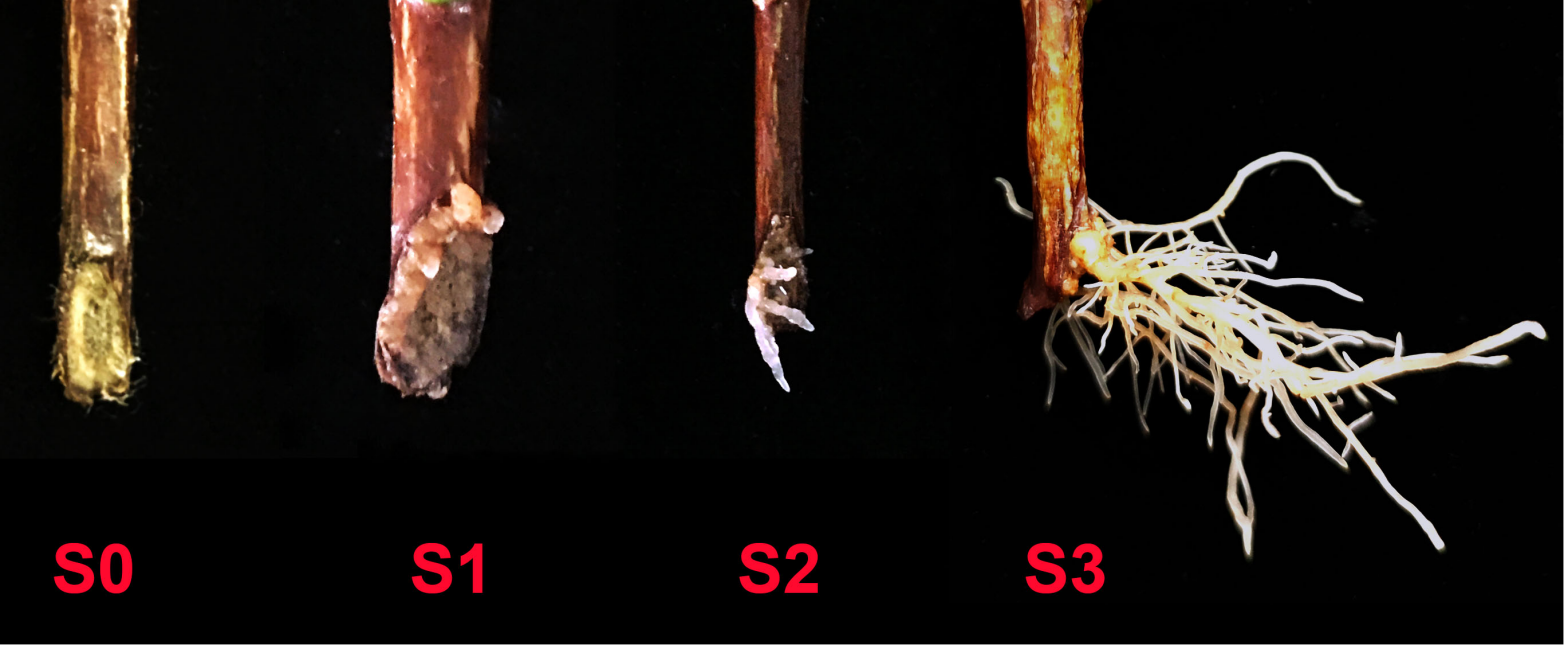
Different rooting stages of tea plant cuttings. S0, S1, S2 and S3 indicated stages of non-rooting, AR protrusion, AR formation and AR elongation, respectively.

The first experiment was sampled at 45 d (days), and the number of cuttings in different rooting stages was recorded. The stages of rooting were as showed in **Figure 1**. To indirectly indicate the existence of AMF in the cutting media, roots of clover were collected for mycorrhizal colonization determination in all treatments. And for treatments in which the cuttings of S3 stage were found, the tea plant roots were also sampled for mycorrhizal colonization determination as direct evidence of successfully colonization of AMF.

The second experiment was sampled at 30 d. The number of cuttings in different rooting stages was also recorded, together with the height and fresh weight of new shoots. Root of clover was used for mycorrhizal colonization determination. Meanwhile, the stem bases (about 0.5 cm) of different rooting stages were sampled and quickly frozen with liquid nitrogen, which were stored in refrigerator at -80°C and used for RNA-Seq analysis and IAA (indole-3-acetic acid) content determination. Cutting in S3 stage was not found in this experiment, so 6 treatments were generated, namely CS0, CS1, CS2, TS0, TS1 and TS2, in which ‘C’, ‘T’ and ‘S’ represented of ‘non-mycorrhizal treatment’, ‘mycorrhizal treatment’ and ‘stage of rooting’, respectively. Each treatment consisted of 3 replicates with about 3-5 tea plant cuttings in each replicate, indicating that there were 18 samples in total.

### Determination of mycorrhizal colonization and indole-3-acetic acid content

As previously described in Chen et al. (2021), mycorrhizal staining was performed according to Phillips and Hayman (1970). Specifically, for tea plant roots, root segments soaked in 20% KOH (w/v) were incubated at 90°C for 30 min in a water bath, then rinsed with tap water and bleached with alkaline hydrogen peroxide (10% H_2_O_2_ + NH_4_OH) for 15 min, followed by acidifying in 5% acetic acid for 5 min, and staining with 0.05% Trypan blue in lactoglycerol (lactic acid:glycerol:water, v:v:v = 1:1:1) at 90°C for 60 min. For clover roots, 20% KOH was changed into 2%, and the temperature was down to 60°C. The procedure of bleaching was also omitted. Then all stained roots were transferred into the destaining solution (lactic acid:glycerol, v:v = 1:1) for 24 h and mycorrhizal colonization was quantified according to Biermann and Linderman (1981).

The content of indole-3-acetic acid (IAA) in basal stems of S1 and S2 in the second experiment were determined by high performance liquid chromatography (HPLC) analysis (Yang et al., 2014). Briefly, about 0.2 g of sample was ground in mortar by adding liquid nitrogen and homogenized with 1.5 mL pre-cooled 80% methanol, which then transferred to 2 mL centrifuge tubes. The samples were extracted at 4°C overnight. After centrifugation at 8,000 g for 10 min at 4°C, the supernatant was collected. The residue was extracted again for 2 h with adding 0.5 mL pre-cooled 80% methanol and supernatant was obtained after centrifugation. All the two parts of supernatant were combined and concentrated to the aqueous phase with nitrogen blowing at 40°C. After decolorization of the extract by adding 2 mL of petroleum ether for three times, an appropriate amount of 1 mol·L^-1^ citric acid was added to the aqueous phase to adjust the pH to 2-3. 2 mL of ethyl acetate was added to extract twice, and then the upper organic phase was transferred to a new centrifuge tube. After drying with nitrogen blowing, the extract was dissolved and mixed in 0.2 mL of mobile phase (1% acetic acid: methanol = 55:45, v:v), and then filtered through a needle filter (0.22 μm) before measuring. The extracts were separated and quantified by HPLC system consisted of a Waters 2695 Separations Module and a Waters 2475 Fluorescence Detector (Waters, Milford, MA, USA). Detection was done with an excitation wavelength at 275 nm and an emission wavelength at 345 nm. Chromatographic conditions were as follows: an Rigol Compass C18 (2) Reversed-Phase column (250 * 4.6 mm, 5 μm) was used in analysis and Mobile phases were 1% acetic acid in water:methanol = 55:45 (V:V). Flow rate was 1 ml·L^-1^ and column temperature was set at 30°C. The injection volume was10 μL. The contents of IAA in all samples were calculated by using the standard curve.

### RNA-seq and transcriptomic analysis and quantitative real-time PCR analysis

Total RNA in the basal stems of tea cuttings from all treatments with 3 independent replicates in each treatment was isolated with the commercial RNA extraction kit from Accurate Biotechnology Co., Ltd, Hunan, China as indicated by the manufacturer’s protocol. Then the RNA integrity was checked with Agilent 2100 Bioanalyzer by using Agilent RNA 6000 Nano Kit and RNA quantification was conducted by Denovix DS-11 spectrophotometer (Denovix Inc., USA). After confirming the RNA quality, one aliquot was sent to Tsingke Biotechnology Co., Ltd (Beijing, China) for RNA-seq by using the Illumina Hiseq™ 4000 platform and 150 bp paired-end reads were generated. Clean reads were obtained for all subsequent analysis by removing reads containing adapters or more than 5% N (N represents base that could not be determined) and low-quality reads from the raw reads. Directly downloaded from the Tea Plant Information Archive (TPIA, http://tpia.teaplants.cn/), the *Camellia sinensis* reference genome data (Wei et al., 2018) were used for the mapping of clean reads by HISAT (Kim et al., 2015). To discover the differentially expressed genes (DEGs) in different comparison sets, DESeq2 (Love et al., 2014) was performed with Q value ≤ 0.05 after aligning clean reads to reference sequences by Bowtie2 (Langmead and Salzberg, 2012) and calculating the expression levels of genes by StringTie (Pertea et al., 2015). Gene ontology (GO) and KEGG (Kyoto encyclopedia of genes and genomes, http://www.genome.jp/kegg/) pathways enrichment analysis of DEGs was implemented by the Blast2GO software (Conesa et al., 2005) and KOBAS 2.0 software (http://kobas.cbi.pku.edu.cn/) (Xie et al., 2011), respectively.

To conduct quantitative real-time PCR (qRT-PCR), another aliquot of extracted RNA was used according to the former established protocol (Chen et al., 2023). In tea plants, *18s rRNA* (*AB120309.1*) was chosen as the reference gene for qRT-PCR analysis as reported by Wei et al. (2013). All primers as showed in **Supplementary Table 1** of randomly selected genes, designed by the Primer 3 software to amplify 150-200 bp fragments, were synthesized in Tsingke Biotechnology Co., Ltd (Beijing, China).

### Statistical analysis

All data were presented as mean ± standard error of three replicates. The independent sample t-test was conducted for two treatments. And for three or more treatments, differences were examined by one-way analysis of variance (ANOVA), and means were compared using with Duncan’s multiple-range test. The analysis was all performed by the IBM SPSS v.25 statistical software (SPSS Inc., Chicago, IL). Additionally, in order to explore the relationship among rooting status, AMF inoculation and auxin-related DEGs, correlation analysis was conducted by using package ‘corrplot’ in R language (Version 4.2.3).

## Results

### Mycorrhizal colonization

In the first experiment, we found that rooting type of tea cuttings in all three varieties (‘Pingyangtezao’, ‘Longjing 43’ and ‘Longjingchangye’) was the comprehensive rooting type, which consists of callus rooting type and cortex rooting type (**Supplementary Figure 1**).

**Figure 2:**
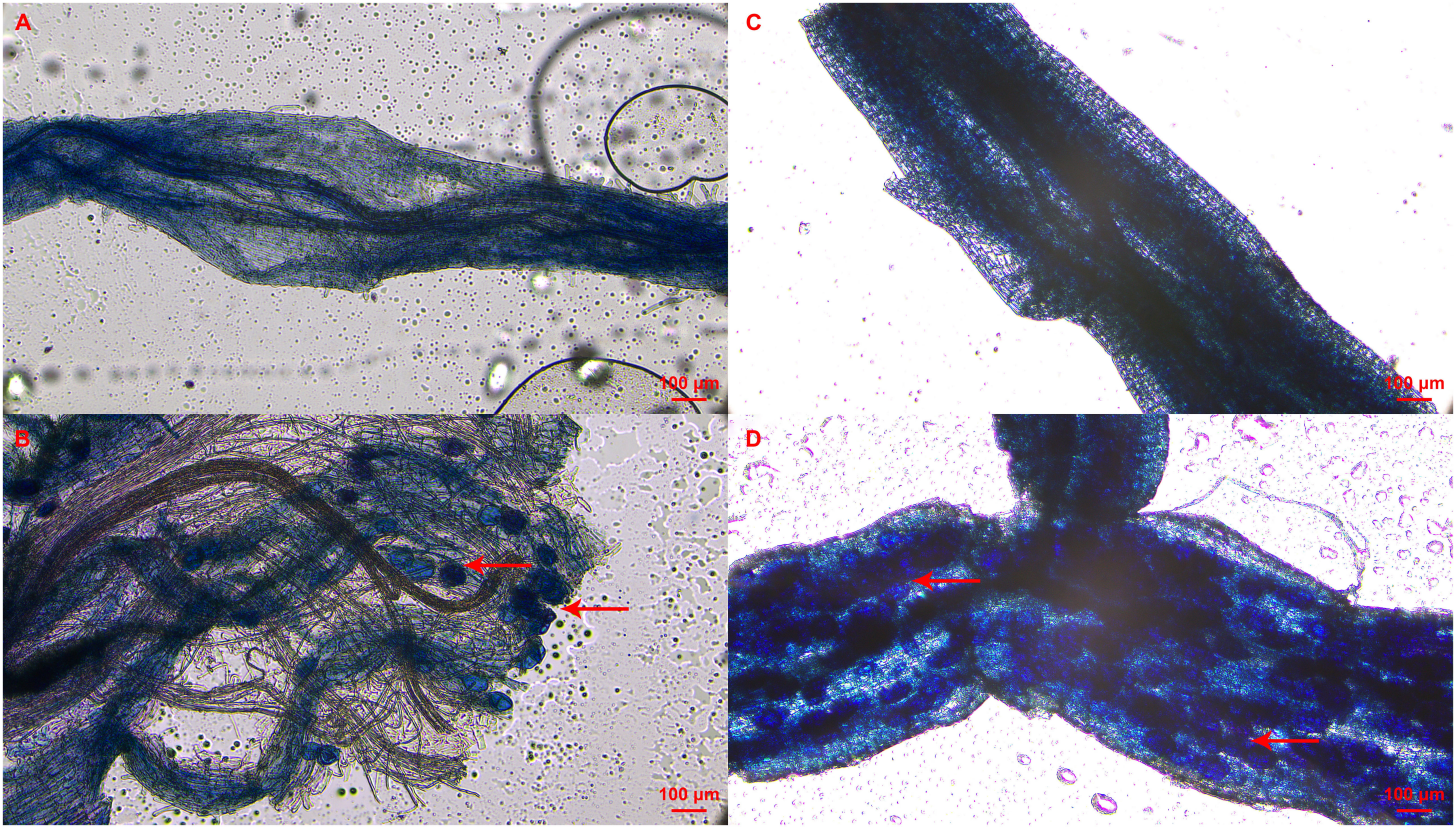
Status of mycorrhizal colonization in clover and tea plant roots at S3 stage.**(A–D)** indicated non-mycorrhizal clover root, mycorrhizal clover root, non-mycorrhizal tea root and mycorrhizal tea root, respectively; Red arrow pointed to the vesicle of AMF.

The mycorrhizal colonization of clover roots was detected to prove the presence of AMF in the cutting substrate. In addition, mycorrhizal colonization of roots in tea cuttings at S3 stage were also determined depending on the rooting status of each tea plant variety. As shown in **Figure 2**, vesicle structures were found in the roots of the inoculated clover and tea cuttings at S3 stage, indicating that AMF was successfully in symbiosis with the plant roots. No AMF structure was found in all non-inoculated treatments (**Figure 2**). In treatment of T and T+IBA, the mycorrhizal colonization of clover roots can reach about 60%, and IBA treatment promoted the colonization of AMF. After treating with different auxin inhibitors, the mycorrhizal colonization has decreased with varying degrees, which decreased the most in the TIBA treatment with less than 40% (**Table 2**). In T and T+IBA treatments, the mycorrhizal colonization of root (S3 stage) in tea cuttings of ‘Pingyang Tezao’, ‘Longjing 43’ and ‘Longjingchangye’ showed a downward trend, but all reached more than 30% (**Table 2**). Similar to the results of clover, IBA also promoted the inoculation of AMF in tea plant roots, while TIBA inhibited it. In AOPP and PEO-IAA treatments, the mycorrhizal colonization decreased slightly with no significance (**Table 2**). Additionally, we also found vesicles in clover roots in the second experiment (**Supplementary Figure 2**) and high mycorrhizal colonization (62.64%) indicated the existence of AMF in the cutting substrate to ensure the accuracy of this research (**Supplementary Table 2**).

**Table 2:** Mycorrhizal colonization in clover and tea plant roots at S3 stage in different treatments.

Treatment	Mycorrhizal colonization (%) in experiments with different tea varieties					
	‘Pingyangtezao’		‘Longjing 43’		‘Longjingchangye’	
	Clover root	Tea plant root at S3	Clover root	Tea plant root at S3	Clover root	Tea plant root at S3
C	0.00 ± 0.00e	0.00 ± 0.00	0.00 ± 0.00d	0.00 ± 0.00b	0.00 ± 0.00e	N/A
T	60.45 ± 4.10ab	37.58 ± 2.80b	62.28 ± 3.58a	33.95 ± 2.21a	55.08 ± 1.49b	31.79 ± 2.57a
C+IBA	0.00 ± 0.00e	0.00 ± 0.00	0.00 ± 0.00d	0.00 ± 0.00b	0.00 ± 0.00e	0.00 ± 0.00b
T+IBA	63.89 ± 1.98a	46.29 ± 2.18a	65.29 ± 0.86a	36.67 ± 2.69a	61.68 ± 3.23a	35.90 ± 4.13a
C+TIBA	0.00 ± 0.00e	N/A	0.00 ± 0.00d	N/A	0.00 ± 0.00e	N/A
T+TIBA	35.39 ± 1.68d	N/A	38.60 ± 2.87c	N/A	39.56 ± 3.67d	N/A
C+AOPP	0.00 ± 0.00e	N/A	0.00 ± 0.00d	N/A	0.00 ± 0.00e	N/A
T+AOPP	48.03 ± 3.37c	N/A	47.56 ± 1.26b	N/A	46.57 ± 2.11c	N/A
C+PEO-IAA	0.00 ± 0.00e	N/A	0.00 ± 0.00d	N/A	0.00 ± 0.00e	N/A
T+PEO-IAA	53.01 ± 5.94bc	N/A	50.77 ± 3.10b	N/A	47.58 ± 2.64c	N/A

N/A represented less or no root in the S3 stage, which was not enough to detect mycorrhizal colonization; C, non-mycorrhizal treatment; T, mycorrhizal treatment; IBA, exogenous auxin; TIBA, inhibitor of auxin transport; AOPP, inhibitor of auxin biosynthesis; PEO-IAA, inhibitor of auxin signal transduction; S3: AR elongation stage; Different letters in the same column represented significant differences at *P* < 0.05 level.

### Rooting status and indole-3-acetic acid content of tea plant cuttings

**Figure 3:**
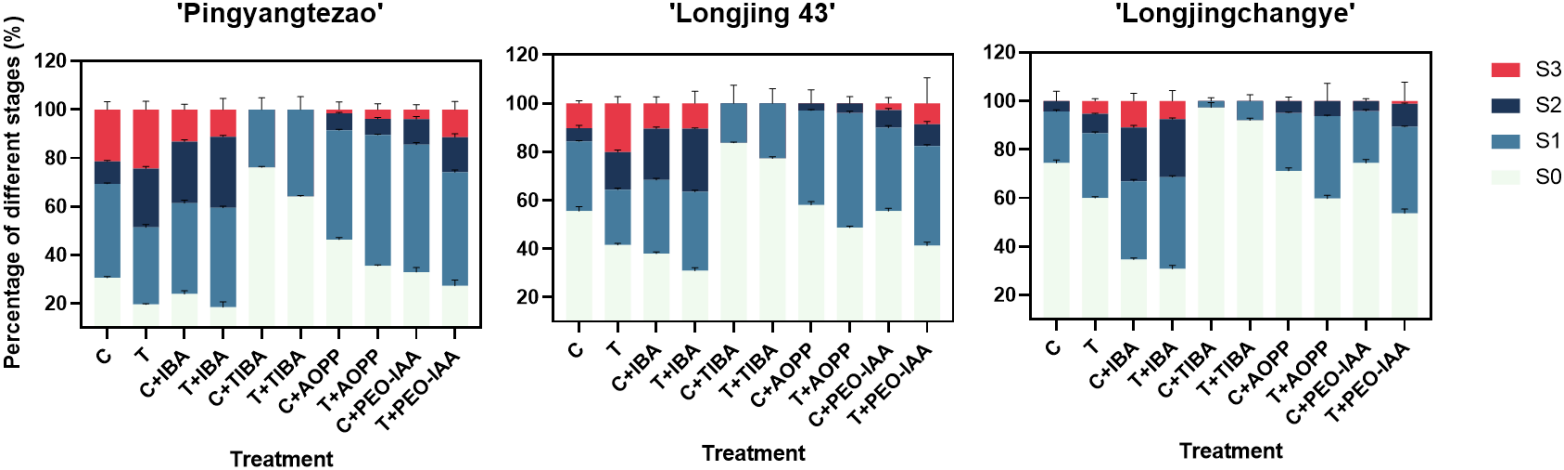
Proportion of tea cuttings at different stages in different varieties. C, non-mycorrhizal treatment; T, mycorrhizal treatment; IBA, exogenous auxin; TIBA, inhibitor of auxin transport; AOPP, inhibitor of auxin biosynthesis; PEO-IAA, inhibitor of auxin signal transduction; S0, S1, S2 and S3 indicated stages of non-rooting, AR protrusion, AR formation and AR elongation, respectively.

**Figure 4:**
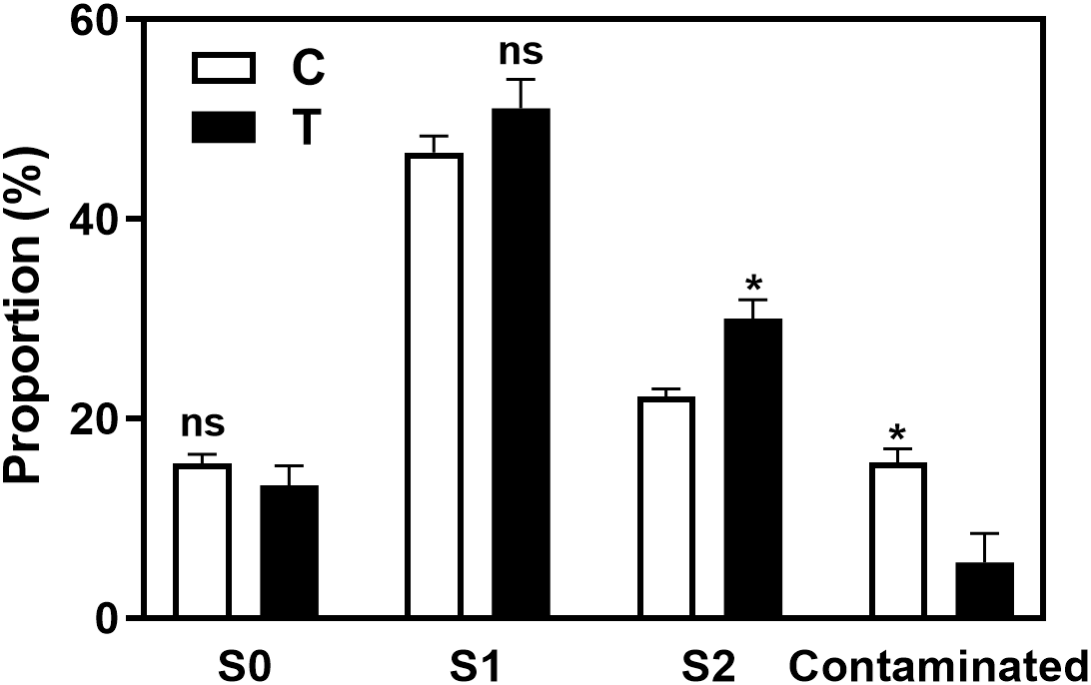
Proportions of contaminated tea plant cuttings and cuttings in different stages in the second experiment. C, non-mycorrhizal treatment; T, mycorrhizal treatment; S1, and S2 indicated stages of AR protrusion and AR formation, respectively. ‘*’ indicated significant difference at *P* < 0.01 level. ‘ns’ indicated no significant difference.

As shown in the **Figure 3** and **Supplementary Figure 3A**, the rooting rate of ‘Pingyang Tezao’ was more than 80% under the T and T+IBA treatments, while in the TIBA treatment, it dropped sharply, although the inoculation treatment could alleviate the inhibitory effect of TIBA on rooting to a extent. The application of auxin synthesis (AOPP) and signal transduction (PEO-IAA) inhibitors also reduced the rooting rate with varying degrees. Under TIBA treatment, the proportion of tea cuttings at S0 stage was the highest, which was 76.14% and 64.16% in C+TIBA and T+TIBA treatments, respectively. The proportion of this stage was the lowest in T+IBA treatment. Compared with C treatment, inoculation with AMF (T) significantly increased the proportion of cuttings at the S2 stage, but after using IBA, there was no significant difference between the two treatments (C+IBA and T+ IBA). AMF had no significant effect on the proportion of cuttings at S1 and S3 stages. The results indicated that AMF may play an important role at S2 stage in ‘Pingyangtezao’. Similar results also found in the second experiment (**Figure 4**) with significant increase of the proportion of cuttings at S2 stage. Interestingly, AMF significantly reduced contamination rates of tea cuttings (**Figure 4**).

As for tea plant variety ‘Longjing 43’, the rooting rate increased to a certain extent, but it did not reach a significant level compared with the corresponding control treatment (**Figure 3** and **Supplementary Figure 3B**). Under T+IBA treatment, the rooting rate was the highest with 69.08%. After TIBA treatment, the rooting rate was significantly lower than other treatments. Compared with C treatment, AMF inoculation (T) significantly increased the proportion of cuttings at S2 (15.49%) and S3 (20.13%) stages. Under T+IBA treatment, the proportion of cuttings at S2 stage was the highest (25.98%). Under TIBA and AOPP treatments, no cuttings at S3 stage were found.

In C+IBA and T+IBA treatments, the rooting rate of ‘Longjingchangye’ was significantly higher than other treatments, which was 65.35% and 69.23%, respectively (**Figure 3** and **Supplementary Figure 3C**). The rooting rate under T treatment (40.04%) was significantly higher than that under C treatment (25.64%). After PEO-IAA treatment, the rooting rate in the control (28.84%) was significantly lower than that in the inoculation treatment (40.31%), indicating that AMF may improve the auxin signal transduction pathway to facilitate the occurrence of adventitious roots. In IBA treatment, regardless of whether AMF was inoculated or not, the proportion of cuttings at S0 stage was significantly lower than that of other treatments. In T treatment, the proportion of cuttings at S3 stage was significantly higher than that in C treatment. AMF also had a certain promoting effect on the proportions of cuttings at S1 and S2 stages but with no significant difference. Except for T+PEO-IAA treatment, no cuttings at S3 stage were observed by using other auxin inhibitors (**Figure 3** and **Supplementary Figure 3C**).

**Figure 5:**
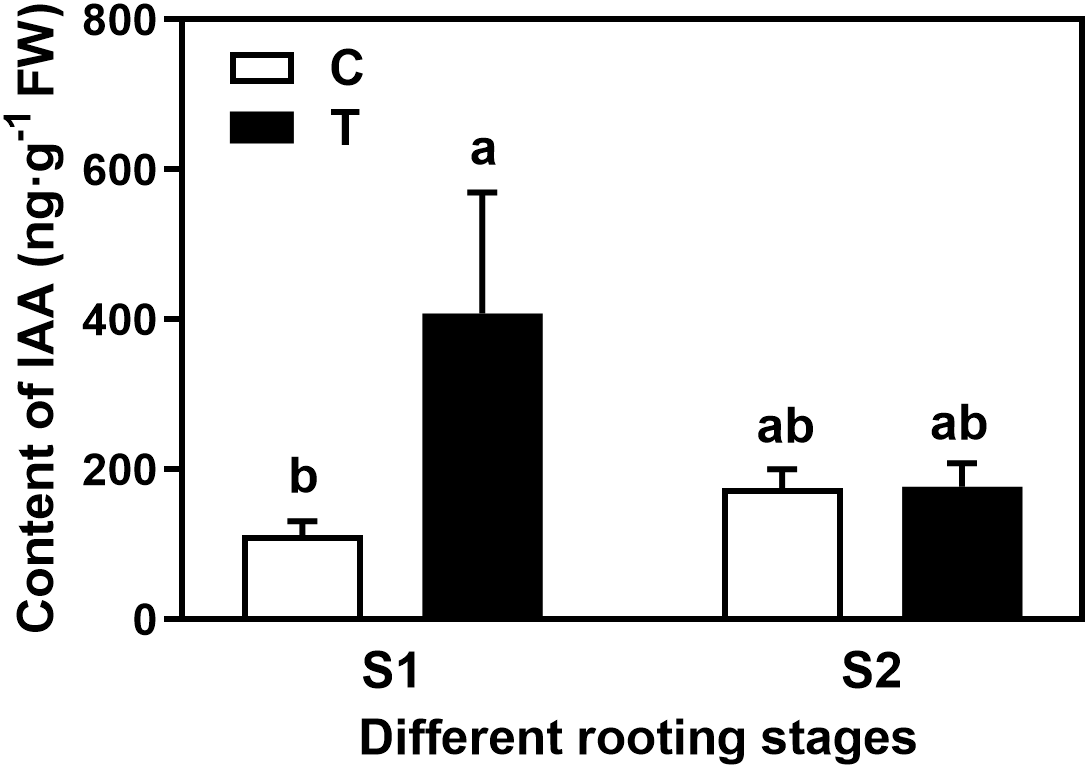
Effect of AMF on the auxin content in the basal stem of tea cuttings. C, non-mycorrhizal treatment; T, mycorrhizal treatment; S1, and S2 indicated stages of AR protrusion and AR formation, respectively. Different letters represented significant differences at *P* < 0.05 level.

Overall, the rooting abilities of the three tea plant varieties from strong to weak were: ‘Pingyangtezao’, ‘Longjing 43’ and ‘Longjingchangye’ as showed in **Figure 3** and **Supplementary Figure 3**. The rooting of ‘Longjingchangye’ seemed more dependent on exogenous IBA than the other two varieties. Variety difference did not affect the regulation of AMF on rooting ability, indicating that AM fungal influence on the occurrence of adventitious root may be independent of variety. Additionally, in the second experiment (‘Pingyangtezao’ as the plant material), proportion of cuttings at S2 stage was significantly increased by AMF inoculation (**Figure 4**). The fresh weight of new shoots in mycorrhizal treatment was lower than that of the control treatment (**Table 3**), suggesting that AMF may promote the accumulation of carbohydrates in the underground part of the cuttings to provide a certain nutritional basis for root development and thus it can penetrate into the root for carbon. We also measured the IAA content of the base stems at S1 and S2 stages (**Figure 5**). Results showed that compared with the control, AMF significantly promoted the IAA content at S1 stage, but no significant difference was found at S2 stage.

**Table 3:** The effect of AMF on the growth of new shoot in tea plant cuttings. ‘C’, ‘T’ and ‘S’ represented of ‘non-mycorrhizal treatment’, ‘mycorrhizal treatment’ and ‘stage of rooting’, respectively.

Treatments	Length of new shoot (cm)	Fresh weight of new shoot (g)
CS0	3.42 ± 0.58 a	0.26 ± 0.06 ab
CS1	4.39 ± 0.90 a	0.36 ± 0.09 a
CS2	4.17 ± 0.50 a	0.33 ± 0.05 a
TS0	2.93 ± 0.49 a	0.14 ± 0.03 b
TS1	3.62 ± 0.39 a	0.21 ± 0.03 ab
TS2	3.40 ± 0.33 a	0.13 ± 0.02 b

Different letters in the same column indicated significance at *P* < 0.05.

### Bioinformatic analysis of RNA sequencing data and identification of differentially expressed genes in stem bases of tea plant cuttings

After sequencing quality control, a total of 121.65 Gb Clean data was obtained, and the percentage of ≥Q30 clean bases of each sample was not less than 93.40% (**Supplementary Table 3** **)**. The Raw data obtained by RNA sequencing (RNA-Seq) in this experiment have been uploaded to NCBI and stored in the PRJNA987374 project (SRA; http://www.ncbi.nlm.nih.gov/sra). According to the statistics of the blast results, the mapping rate of clean reads of each sample to the reference genome was above 75%, except for the two biological replicates of CS02 and TS02, which had a relatively low mapping rate (**Supplementary Table 3**). The number of differentially expressed genes (DEGs) in different comparison sets of CS0 vs. TS0, CS1 vs. TS1 and CS2 vs. TS2 was 202, 393 and 1360 (**Table 4**), that is to say, with the progress of root development, the greater effect of AMF on the tea cuttings was found. The results also indicated the stronger regulation of AMF on tea cuttings at S2 stage (**Table 4**). As showed in **Supplementary Data 1**, 1 (*LOC114297066*) and 2 DEGs (*LOC114258175* and *LOC114297066*) related to pectinesterase in most 10 up-regulated DEGs of CS1 vs. TS1 and CS2 vs. TS2, respectively. Interestingly, 3 DEGs related to peroxidase (*LOC114290111*, *LOC114291213* and *LOC114266646*) were among the most 10 up-regulated DEGs in CS2 vs. TS2. These genes may deserve more attentions in the future. Additionally, although *WUSCHEL-RELATED HOMEOBOX GENE 11* (*WOX11*) plays a critical role in adventitious root formation and development, in our results only *WUSCHEL-related homeobox 11-like* (*LOC114291237*) was significantly up-regulated by AMF in DEGs of CS2 vs. TS2, which may indicate the minor effect on this gene of AMF.

**Table 4:** Results of statistical analysis of differentially expressed genes. C, non-mycorrhizal treatment; T, mycorrhizal treatment; S0, S1, and S2 indicated stages of non-rooting, AR protrusion, and AR formation, respectively.

Comparison set	Total DEGs	Up	Down
CS0 vs. TS0	202	137	65
CS1 vs. TS1	393	232	161
CS2 vs. TS2	1360	954	406

The validation of the RNA-Seq data was also conducted by real-time quantitative PCR (qRT-PCR) in order to take further analysis. 11 genes, including phytohormone-related genes, were randomly selected for qRT-PCR. Results showed that the same trend was found between the expressions identified by qRT-PCR and FPKM values (Fragments Per Kilobase of exon model per Million mapped fragments) in RNA-Seq (**Supplementary Figure 4**). Moreover, a significantly positive correlation (R^2 = ^0.8695, *P* < 0.01, **Supplementary Figure 5**) between RNA-Seq and qRT-PCR data indicated that the RNA-Seq data were credible for exploring the mechanism of AM fungal regulation on the adventitious root formation of tea cuttings.

### Functional classification of differentially expressed genes

As showed in **Supplementary Data 2**, the first three GO (Gene Ontology) terms with the most enriched genes in all comparison sets (CS0 vs. TS0, CS1 vs. TS1 and CS2 vs. TS2) were ‘metabolic process’, ‘cellular process’ and ‘single- organism process’ in the category of ‘Biological process’. With the proceed of rooting of tea cuttings, the enriched genes also increased (**Supplementary Data 2**). Similarly, in the ‘Cellular component’ and ‘Molecular function’ categories, ‘membrane’ and ‘catalytic activity’ were GO terms with the most enriched genes in the three comparison sets, respectively. Noteworthily, GO term of ‘antioxidant activity’ enriched 4, 4, and 33 DEGs in the CS0 vs. TS0, CS1 vs. TS1, and CS2 vs. TS2 comparison sets, respectively, indicating that in the S2 stage of rooting, AMF might exert a greater impact on the antioxidant system (**Supplementary Data 2**).

As for results of KEGG analysis, pathway of ‘Plant hormone signal transduction’ was induced by AMF treatment in all three comparison sets, which was significantly different from the control treatment (**Supplementary Data 2**), indicating that AMF may affect rooting of tea cuttings by regulating plant hormone-related pathways. Pathway of ‘Phenylpropane biosynthesis’ was significantly enriched in CS2 vs. TS2 (**Supplementary Data 2**), which was related to the well-enriched GO term of ‘antioxidant activity’. In addition, pathways of ‘Glutathione metabolism’, ‘Amino sugar and nucleotide sugar metabolism’ and ‘Plant-pathogen interaction’ were also affected by AMF inoculation in all comparison sets. In CS1 vs. TS1 and CS2 vs. TS2, both 7 DEGs were enriched in pathway of ‘Starch and sucrose metabolism’, indicating the AM fungal regulation on the carbon metabolism in these two stages. Interestingly, pathway of ‘Circadian rhythm’, which is generally related with the adventitious root induction, was enriched with 6 DEGs in CS0 vs. TS0, followed by 3 DEGs in CS2 vs. TS2 and 1 DEGs in CS1 vs. TS1. The result showed that AMF may affect this pathway mainly in the S0 and S2 stage of tea cuttings (**Supplementary Data 2**).

### Differentially expressed genes pertaining to different pathways of auxin

**Figure 6:**
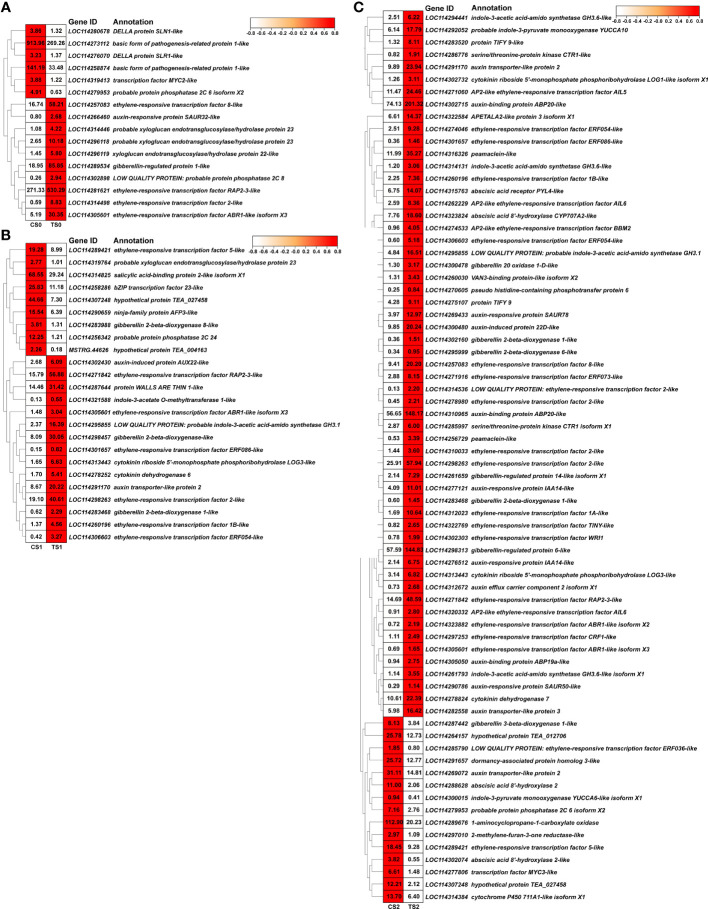
DEGs related to plant hormone in different sets of CS0 vs. TS0 **(A)**, CS1 vs. TS1 **(B)** and CS2 vs. TS2 **(C)**. C, non-mycorrhizal treatment; T, mycorrhizal treatment; S0, S1, and S2 indicated stages of non-rooting, AR protrusion, and AR formation, respectively.

The above KEGG results showed that phytohormone-related pathway may play an important role in the regulation of AR formation in tea cuttings by AMF. Therefore, DEGs (Differentially expressed genes) pertaining to phytohormones (especially auxin) were excavated out based on the annotation results by using KEGG and Nr databases, and relevant results were showed in **Supplementary Data 3** and **Figure 6**. There were 16 and 24 phytohormone-related DEGs at S0 and S1 stages of tea cuttings induced by AMF inoculation, respectively, while AMF had the most obvious regulation on root development at S2 stage (72 DEGs, **Supplementary Data 3**). Specifically, number of phytohormone-related DEGs regulated by AMF was obvious increased (72 DEGs), and almost all auxin-related genes (17 of 21 DEGs) were significantly up-regulated (**Supplementary Data 3**), including genes related to auxin biosynthesis and metabolism (*LOC114292052*, *LOC114295855*, *LOC114294441, LOC114314131*, *LOC114261793*), transport (*LOC114291170*, *LOC114282558*, *LOC114312672*) and signal transduction (*LOC114302715*, *LOC114300480*, *LOC114269433*, *LOC114310965*, *LOC114290786*, *LOC114305050*, *LOC114277121*, *LOC114276512*) as showed in **Figure 6**.

### Correlation analysis among rooting rate, AMF inoculation and auxin-related differentially expressed genes

**Figure 7:**
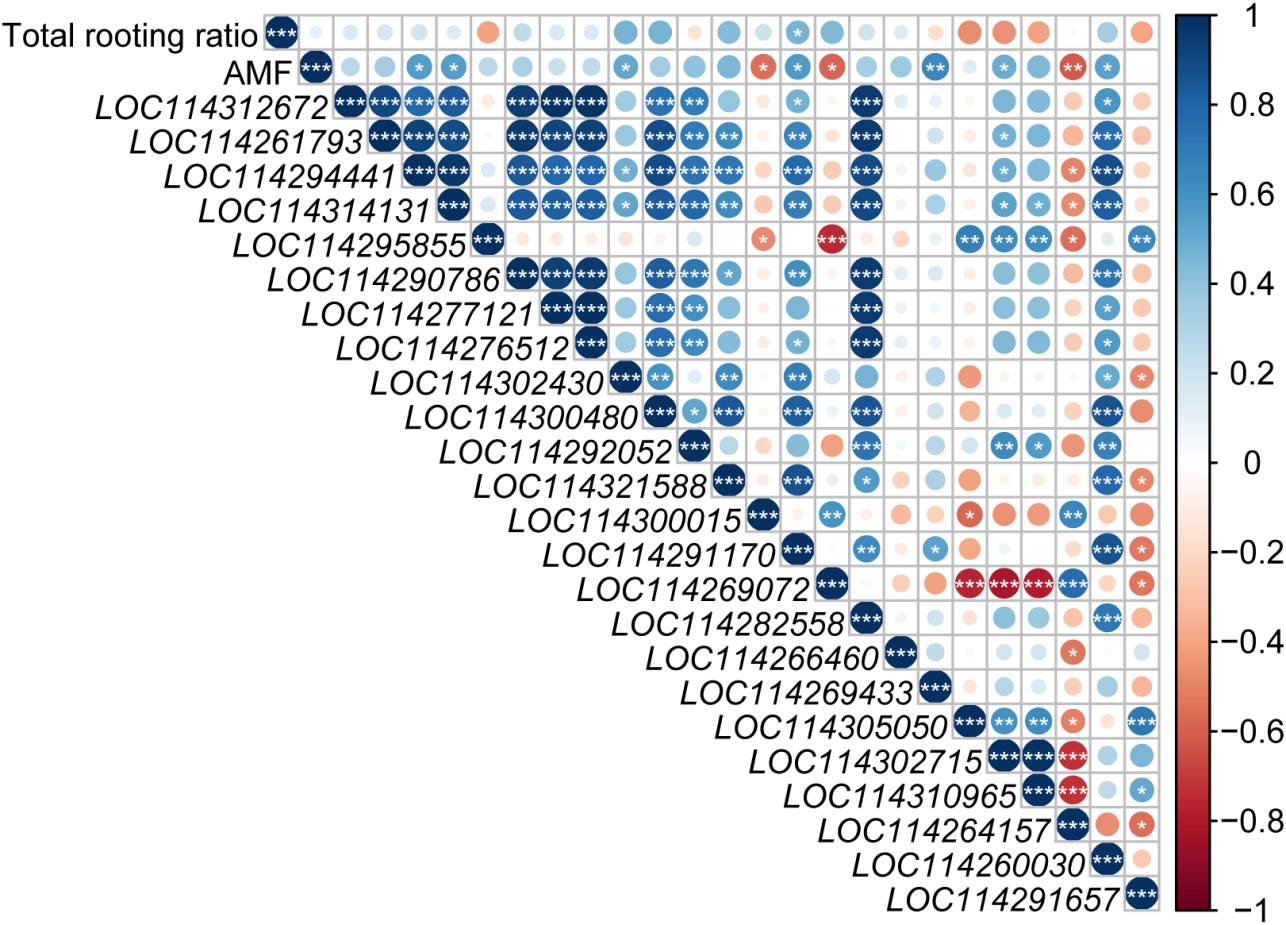
Analysis of the relationship among auxin-related genes, rooting rate and AMF inoculation. Blue and red circle indicated positive and negative correlation, respectively. ‘*’, ‘*’ and ‘***’ in the circles indicated the significant difference at *P* < 0.05, *P* < 0.01 and *P* < 0.001, respectively. No significant difference was marked with no star.

Total rooting ratio was significantly positively correlated with *LOC114291170* (*auxin transporter-like protein 2*, *LAX2*) expression (**Figure 7**). AMF inoculation was significantly positively correlated with expressions of *LOC114294441* (*Indole-3-acetic acid-amido synthetase GH3.6-like*), *LOC114314131* (*Indole-3-acetic acid- amido synthetase GH3.6-like*), *LOC114302430* (*auxin-induced protein AUX22-like*), *LOC114291170* (*auxin transporter-like protein 2*, *LAX2*), *LOC114302715* (*auxin-binding protein ABP20-like*), *LOC114269433* (*auxin-responsive protein SAUR78*) and *LOC114260030* (*VAN3-binding protein-like isoform X2*, auxin canalization related gene), but negatively with *LOC114300015* (*Indole-3-pyruvate monooxygenase YUCCA6-like isoform X1*), *LOC114269072* (*auxin transporter-like protein 2*, *LAX2*), *LOC114264157* (*hypothetical protein TEA_012706*, *AUX/IAA family*).

## Discussion

Cutting propagation has always been the main measure for the promotion and application of excellent varieties of tea plant (*Camellia sinensis* L.), which is conducive to maintaining the excellent characteristics of varieties. Compared with other difficult-to-root woody plants, tea plant cuttings exhibit a lower degree of difficulty for rooting. One of the reasons is that the adventitious rooting type in most varieties of tea plant is the comprehensive rooting type, and the cuttings with more cortex rooting type are easier to root (Ma et al., 2015; Liu, 2017). Our findings in this paper also demonstrated this as well (**Supplementary Figure 1**). However, in the comprehensive rooting type, the proportion of cuttings of callus rooting and cortex rooting type in various varieties was not recorded in this experiment, and further research can be carried out in the future.

Adventitious root (AR) formation is the most critical step in cutting propagation, and a good root system architecture is crucial to the survival rate and resistance of seedlings to various stresses after transplanting (Chen et al., 2016). Previous studies have shown that arbuscular mycorrhizal fungus (AMF), which can form a symbiotic relationship with the roots of most terrestrial plants, has great potential to affect plant cutting propagation (Ávila Díaz-Granados et al., 2009; Krishna et al., 2013; Essahibi et al., 2017; Fernades et al., 2019) and construction of root system architecture (Chen et al., 2016), however, there are still few studies on the effect of AMF on the rooting of tea cuttings (Liu and Lei, 1995; Gao et al., 2023), which mainly focused on the growth of tea seedlings in the later stage without covering the regulatory mechanism of adventitious root formation induced by AMF in the early stage. Therefore, in this paper, in order to decipher the relative mechanism, we divided the rooting state of tea cuttings into four stages for the first time (**Figure 1** **)**: S0 (stage of non-rooting), S1 [stage of adventitious root (AR) protrusion], S2 (stage of AR formation) and S3 (stage of AR elongation). Moreover, the cutting samples of these four rooting states were obtained at the same time to accurately clarify the mechanism of AMF affecting adventitious rooting. In addition, previous studies have shown that auxin plays a key role in the process of AR development, therefore, we also studied the effect of combined treatment of auxin and AMF on rooting of tea cuttings, and different auxin inhibitors were first applied to reveal AM fungal regulation of auxin-related pathways during rooting in cuttings.

### Rooting ability regulated by Arbuscular mycorrhizal fungus and auxin in tea cuttings

AMF successfully established a symbiotic relationship with roots of clover and tea cuttings at the S3 stage (**Table 2**; **Supplementary Table 2**; **Figure 2**; **Supplementary Figure 2**), and the mycorrhizal colonization of the tea plant root at S3 stage reached more than 30% (**Table 2**) with the highest in variety of ‘Pingyangtezao’, indicating that the inoculation efficiency of AMF in tea plant root may depend on the variety. In addition, *Arbuscular Mycorrhization 2* (*RAM2*) encodes a glycerol- 3-phosphate acyltransferase and is necessary for transferring lipids from plants to AMF together with undertaking a “signaling” role on the root surface (Jiang et al., 2017; Liu et al., 2022). Localized in the periarbuscular membrane, *STR* (*Stunted Arbuscule* genes) and *STR2* encode ATP-binding cassette transporters, which are also responsible for lipid transfer to AMF and indispensable for arbuscule formation (Gutjahr et al., 2012; Jiang et al., 2017; Feng et al., 2020). It was found that these mycorrhizal colonization related genes, *RAM2* (*LOC114297081*), *STR* (*LOC114323273*) and *STR2* (*LOC114276941*), were only significantly up-regulated in the cuttings at the S2 stage (**Supplementary Data 1**) compared with the control in the second experiment of this study. And it was supposed that ARs have started to establish mycorrhizal symbiosis with AMF, which is consistent with our previous observations in the roots of mycorrhizalized tea seedlings (Chen et al., 2023). All these results could indicate that the presence of AMF in the cutting substrate or root of cuttings, making it possible to further analyze how AMF affects the rooting of tea cuttings.

Overall, AMF promoted adventitious root formation and rooting rate of tea cuttings (**Figures 2** and **4**; **Supplementary Figure 3**), which was consistent with finding of previous studies in herbaceous (Thanuja et al., 2002; Sohn et al., 2003; Fernades et al., 2019) and woody plants (Han et al., 2013; Amri, 2015; Essahibi et al., 2017). Treatment of exogenous auxin shared similar results with AMF inoculation, both of which increased the rooting rate of cuttings of different tea plant varieties, especially in the ‘Longjingchangye’ variety which was relatively difficult to root according to our results (**Figure 3** and **4**; **Supplementary Figure 3**). The result was in conformity with other related studies in tea cutting propagation with application of auxin (Chai et al., 2015; Ren et al., 2019; Wang and Gong, 2019). However, auxin-related inhibitors applied in this paper directly reduced the rooting rate, and the effect of TIBA treatment was the most significant (**Figure 3** and **Supplementary Figure 3**). Moreover, the combined application of IBA and AMF also had a positive effect on the rooting rate of cuttings (**Figure 3** and **Supplementary Figure 3**), indicating that AMF and auxin can synergistically promote adventitious rooting in tea cuttings, which was confirmed by the decreased rooting ability of tea cuttings when using different auxin-related inhibitors in mycorrhizal treatments. We speculated that this may be related to the effect of exogenous auxin on the symbiosis establishment between AMF and root. In the first experiment, IBA treatment significantly promoted the infection of AMF on the roots of ‘Pingyangtezao’ cuttings (46.29%, **Table 2**), which was higher than our previous mycorrhizal experiments on tea plant (Chen et al., 2021; Chen et al., 2023). Same results were also found in the other two varieties, but did not reach a significant level. After using auxin-related inhibitors, the colonization rate of AMF in clover roots decreased to varying degrees. All these further confirmed the important role of auxin in the process of establishing a symbiotic relationship between AMF and plants, which is found in other studies (Zhang et al., 2018; Ludwig-Müller, 2019; Chen et al., 2022a). For instance, Chen et al. (2022a) found a positive correlation between the IAA content and mycorrhization level, particularly arbuscule incidence.

### Auxin pathways involved in the AM fungal regulation on adventitious root formation of tea cuttings

Considering the role of auxin in AMF symbiosis, it is speculated that auxin-related pathways may also be involved in the process of AMF promoting adventitious root formation in tea cuttings. In the study of blue light-induced adventitious rooting of tea cuttings (Shen et al., 2023), auxin signaling transduction genes, such as *AUX/IAA* (*auxin responsive protein*), *ARF* (*auxin response factor*) and *SAUR* (*auxin-responsive protein*) responded most rapidly (16 h after treatment). Moreover, genes related to auxin transport (*PIN1*, *3* and *4*) and biosynthesis (*YUC9* and *10*) changed with indole-3-carboxylic acid (ICA) content. All these genes are closely related to AR formation of tea cuttings. Therefore, in this paper, we screened out the genes related to different pathways of auxin from the DEGs of the three comparison sets, and found that the number of genes related to auxin pathways of biosynthesis and metabolism, transport and signal transduction was most at S2 stage with 72 DEGs (**Figure 6C** and **Supplementary Data 3**), while at other two stages, the number was only 16 and 24 (**Figures 6A, B**), respectively, which indicated the stronger regulation by AMF on AR formation at S2 stage of tea cuttings.

Among the Trp (tryptophan)-dependent pathways of auxin biosynthesis, *YUCCA* (*YUC*) is a key gene and encodes a flavin monooxygenase, which can catalyzes the oxidative decarboxylation of pyruvic acid (IPA) to form IAA (Li and Li, 2019; Zheng et al., 2023). The irreversible reaction triggered by this gene could be used for regulating auxin contents in plants. In this paper, we only found two *YUC* DEGs (*LOC114300015* and *LOC114292052*) in tea cuttings at S2 stage (**Figure 6C** **;** **Supplementary Data 3**), in which the expression of *YUC10* (*LOC114292052*) induced by AMF inoculation increased about 3 times more than that in the control treatment, indicating that AMF may promote the biosynthesis of auxin in AR which was just formed. Similar results also found in the study of Shen et al. (2023) together with another increased *YUC* gene (*YUC9*). At stage of S0 and S1, no significant expressions of *YUC* genes between mycorrhizal treatment and the control showed the less affection of AMF on auxin biosynthesis of tea cuttings.

*GRETCHEN HAGEN3* (*GH3*) family genes, encoding IAA-amido synthetases, promote auxin to bind to amino acids, which controls the level of auxin in plants (Chen et al., 2010). In comparison sets of CS1 vs. TS1 and CS2 vs. TS2, 1 and 4 *GH3* DEGs were found, respectively (**Supplementary Data 3**), in which *GH3.1* (*LOC114295855*) was both significantly up-regulated by AMF. Three *GH3.6* (*LOC114261793*, *LOC114314131*, *LOC114294441*) genes were all highly expressed at S2 stage. These genes may be involved in the AM fungal regulation on the AR formation of tea cuttings. However, in apple, overexpressing *MdGH3.6* reduced IAA content, AR number, root length and water-deficit stress tolerance (Jiang et al., 2022). Functional analysis of the three *GH3.6* genes need more further study in the future to clarify relative mechanisms. In addition, Chen et al. (2022a) suggested that the finely tuned development of arbuscular mycorrhiza (AM) involves the regulation of expansin genes and auxin homeostasis mediated by *SlGH3.4*, which encodes a putative IAA-amido synthetase. Generally, *SlGH3.4* negatively regulate mycorrhization via maintaining cellular auxin homoeostasis. Even though *GH3.4* was not found in DEGs, AMF inoculation indeed significantly increased the IAA content at the base of tea cuttings at S1 stage (**Figure 5**), which may contribute to the high rooting rate of AR. However, whether *GH3.4* plays a role or not in this process was not clear. Similar results were happened to genes of *CsGH3.2* and *CsGH3.3*, which play negative regulatory roles in the AR development of tea (*Camellia sinensis* cv. Taixuan 0310) cuttings (Chen et al., 2022b). The different results may be due to variety differences.

Auxin in higher plants can be transported in two ways, long-distance vascular transport and short-distance active transport, also known as auxin polar transport (Li and Li, 2019). The latter plays a key role in the asymmetric distribution of auxin and requires transport carriers, which mainly include AUX/LAX family proteins (auxin influx carriers), PIN (PIN-FORMED) family proteins (auxin efflux carriers), and ABCB/MDR/PGP family proteins with both influx and efflux functions (Cho and Cho, 2013; Liu et al., 2023b). Plants generally fulfil the polar transport and distribution of auxin by regulating these family proteins. In the second experiment, 4 DEGs related to auxin transport in total was found in two comparison sets of S1 and S2 stages with no DEGs in CS0 vs. TS0 (**Figure 6** and **Supplementary Data 3**). In CS1 vs. TS1 and CS2 vs. TS2, *LAX2* (*LOC114291170*) was both up-regulated by AMF. Interestingly, there was another *LAX2* gene (*LOC114269072*) down-regulated only in CS2 vs. TS2, while *LAX3* (*LOC114282558*) and *PIN2* (*LOC114312672*) were all induced by inoculation of AMF. Thus, *LAX* and *PIN* genes seemed to participate in the AM fungal regulation on the AR formation for auxin transport in our experiment. *PIN2* determines the direction and extent of cell division in root meristem and the formation of root patterns (Barbosa et al., 2018), and *PtPIN2* is specifically expressed in ARs involved in AR development by regulating the polar auxin transport (Liu et al., 2014), which explained that *PIN2* only expressed at S2 stage in this paper. In addition, *OsPIN2* is involved in the regulation of root architecture, root elongation growth, lateral root formation of rice (Inahashi et al., 2018). After S2 stage, the newly-formed AR will elongate, which required the expression of *PIN2*. Hu et al. (2023) found 8 *PIN* genes in tea plant and confirmed that *PIN3* was involved in the regulation of root growth and development as well as auxin accumulation. But in our results, no significant difference was found between mycorrhizal treatment and the control of the *PIN3* expression. On the other hand, *LAX3*, which was up-regulated in our mycorrhizal treatment at S2 stage, plays a role in mediating adventitious root formation (Swarup and Bhosale, 2019). *LAX2* and *LAX3* are positively correlated with adventitious root number in apple, but negative with the length of AR (Li et al., 2018), which may partially explain that one *LAX2* gene was down-regulated by AMF at S2 stage. Further research will be conducted to clarify the different mechanisms of the two *LAX2* genes in tea cuttings.

Auxin signal transduction controls the response of tissues and organs to auxin and is an important part of plant auxin research. The auxin responsive protein SAUR family, Aux/IAA family, auxin-binding protein (ABP) family, auxin response factor (ARF) family, crucial in the adventitious root formation, are key genes in the auxin signal transduction (Della Rovere et al., 2015; Ayala et al., 2022). In our experiment, 4 (*LOC114302430*, *LOC114260030*, *LOC114302715* and *LOC114269433*) of 7 auxin-related DEGs, which were significantly positively correlated with AMF inoculation, were belong to auxin signal transduction pathway (**Figure 7**), indicating the important role of auxin signal transduction in AM fungal regulation on the rooting of tea cuttings. Different from the number of DEGs related to auxin signal transduction in CS0 vs. TS0 (*LOC114266460*) and CS1 vs. TS1 (*LOC114321588*, *LOC114302430*), there were 11 DEGs in CS2 vs. TS2, among which three *ABP* genes (*LOC114310965*, *LOC114305050* and *LOC114302715*) were all up-regulated by AMF inoculation, as well as *AUX22D* (*LOC114300480*), *SAUR71* (*LOC114290786*) and *IAA14* (*LOC114277121*). With the process of AR formation, regulation of auxin signal transduction pathway was getting more complicated. Moreover, *SAUR*, *Aux/IAA* and *ABP* genes may strongly response to AMF inoculation than other related genes. *SAUR15* can promote adventitious root development of Arabidopsis via auxin accumulation by activating auxin biosynthesis genes (Yin et al., 2020). Previous research also found that in the induction phase of AR production, *SAUR* was mainly up-regulated, but down-regulated in the root primordium formation stage (Ai et al., 2023), and we screened out a *SAUR* gene (*auxin-responsive protein SAUR32-like*, *LOC114266460*) in CS0 vs. TS0. *SAUR71* (*LOC114290786*) also found at S2 stage may indicate the existence of root primordium induction process triggered by AMF. Similar to our results in AMF treatment, *ABP* genes were up-regulated by Nitric Oxide to stimulate the formation of adventitious root (Li et al., 2020). *AUX22* will be up-regulated after auxin treatment (Ayala-Doñas et al., 2022), and two elevated expressions of *AUX22* (*LOC114302430* of S1 stage and *LOC114300480* of S3 stage) genes in our experiment may indicate the increased IAA content in the tea cuttings.

## Conclusions

Tea plants have been cultivated and spread worldwide primarily through cutting propagation, also known as vegetative reproduction, to keep excellent characteristics. Adventitious root (AR) formation, a key step for cutting propagation, was promoted by arbuscular mycorrhizal fungus (AMF) and auxin application in this paper. The use of indolebutyric acid (IBA) increased the mycorrhizal colonization rate and interacted with AMF in mediating rooting ability of tea cuttings. AMF strongly affect the genes at S2 stage of cutting, followed by S1 and S0 stage. *GH*, *PIN*, *LAX*, *SAUR*, *AUX*, and *ABP* genes may play important roles in the AM fungal regulation on AR formation of tea cuttings. All results provided a theoretical basis for propagation of mycorrhizal tea seedlings and AM fungal regulation of adventitious roots.

## Data availability statement

The original contributions presented in the study are included in the article/**Supplementary Files**, further inquiries can be directed to the corresponding author.

## Author contributions

WC: Conceptualization, Investigation, Supervision, Writing – original draft, Writing – review & editing. WS: Investigation, Writing – review & editing. TN: Investigation, Writing – review & editing. TY: Investigation, Writing – review & editing. QS: Investigation, Methodology, Writing – review & editing. JZ: Conceptualization, Writing – review & editing.
