# Supplementary material for: Insight into regulation of adventitious root formation by arbuscular mycorrhizal fungus and exogenous auxin in tea plant (Camellia sinensis L.) cuttings
Source: Front Plant Sci. 2023 Sep 18;14:1258410. doi: 10.3389/fpls.2023.1258410 (PMC10544935; doi:10.3389/fpls.2023.1258410)

Supplementary Material

Title:

Insight into regulation of adventitious root formation by arbuscular mycorrhizal fungus and exogenous auxin in tea plant (*Camellia sinensis* L.) cuttings

Authors:

Weili Chen, Wenshu Shan, Tingting Niu, Tao Ye, Qinyu Sun, Jiaxia Zhang*

*****Correspondence: Jiaxia Zhang: [zhangjiaxia035@163.com](mailto:zhangjiaxia035@163.com)

# Supplementary Tables and Figures

# **Supplementary Table 1** Primers of genes in real-time quantitative RT-PCR

| **Gene ID** | **Gene Annotation** | **Sequence of primers (5’-3’)** |
| --- | --- | --- |
| *LOC114269433* | *auxin-responsive protein SAUR78* | F: TCTTCCACCTCCGTAACAGC  R: CCAGGACTTGGAACAGAGGG |
| *LOC114276125* | *heterodimeric geranylgeranyl pyrophosphate synthase small subunit, chloroplastic-like* | F: AGAGATGGAAGTGGTAGAGAGGT  R: AAGAATCACACACCCTGCCC |
| *LOC114276742* | *high affinity nitrate transporter 2.4-like* | F: GAGGGGTGATGTTCCCAAAGA  R: GTCAGAAGCGTATCCTCCGAA |
| *LOC114279535* | *probable pectinesterase/pectinesterase inhibitor 6* | F2: AAACGGTGGGACGAACAGAT  R2: AAAGCTTACCAACAGTTGCAGAG |
| *LOC114283520* | *protein TIFY 9-like* | F: ATCCAGAACTCGCGTTGTCTT  R: AAATGTGTTGCGGGTTGGAC |
| *LOC114284689* | *lectin beta-1 and beta-2 chains-like* | F: TCAAACCGCTTCACCTTTATGAC  R: GCAAGCCCATCTCCGAAAGT |
| *LOC114288371* | *glutathione S-transferase U17-like* | F: AATCTGATGAAGTGAAAAAGCCTCA  R: CAATAGAAGGGGAAAACTAAGCGA |
| *LOC114295855* | *probable indole-3-acetic acid-amido synthetase GH3.1* | F: TGAAGAGCTGGATCGTCGTC  R: GGCTTCGTTTGGACTTGTGT |
| *LOC114301470* | *pectinesterase 2-like* | F: GTCAGTCGTGGTTGCCTTCT  R: TTCTGGGATTCCCTATGTTTTGTG |
| *LOC114314498* | *ethylene-responsive transcription factor 2-like* | F: AAGAAGGTGGTGGCGAAAGA  R: CTGCGCAACCGATTTTGTCC |
| *LOC114314536* | *ethylene-responsive transcription factor 2-like* | F: AGAAGGTGGTGGCGAAAGTG  R: AGCCCTTGGTCAGAACTCAG |
| *AB120309.1* | *18S rRNA* | F: CGCGCAAATTACCCAATCCT  R: ACCAGACTTGCCCTCCAATG |

# **Supplementary Table 2** Mycorrhizal colonization of clover roots in the second experiment. C, non-mycorrhizal treatment; T, mycorrhizal treatment; ‘**’ indicated a significant difference at *P* < 0.01 level.

| **Treatment** | **Mycorrhizal colonization (%)** |
| --- | --- |
| C | 0.00±0.00 |
| T | 62.64±2.50** |

# **Supplementary Table 3** Main results of RNA-Seq. C, non-mycorrhizal treatment; T, mycorrhizal treatment; S0, S1, and S2 indicated stages of non-rooting, AR protrusion, and AR formation, respectively.

| Samples | Clean reads | GC Content (%) | ≥Q30 clean bases (%) | Mapping rate (%) | Uniq mapping rate (%) |
| --- | --- | --- | --- | --- | --- |
| CS01 | 21,778,793 | 45.27 | 94.71 | 82.87 | 76.85 |
| CS02 | 23,071,591 | 47.92 | 94.37 | 66.50 | 60.91 |
| CS03 | 21,431,228 | 46.27 | 94.56 | 75.03 | 69.26 |
| CS11 | 24,713,336 | 44.72 | 94.16 | 86.97 | 81.00 |
| CS12 | 27,043,406 | 45.28 | 93.83 | 86.95 | 80.09 |
| CS13 | 22,618,534 | 44.70 | 94.62 | 87.95 | 82.00 |
| CS21 | 21,310,190 | 44.69 | 94.41 | 88.92 | 82.95 |
| CS22 | 22,101,963 | 44.80 | 94.46 | 88.66 | 82.50 |
| CS23 | 22,293,840 | 44.77 | 94.68 | 87.98 | 82.01 |
| TS01 | 20,870,581 | 45.97 | 93.40 | 77.80 | 71.62 |
| TS02 | 22,249,504 | 48.67 | 94.42 | 55.06 | 50.38 |
| TS03 | 27,992,867 | 46.08 | 93.90 | 75.66 | 70.29 |
| TS11 | 22,311,822 | 44.81 | 94.33 | 86.81 | 80.79 |
| TS12 | 17,364,002 | 44.99 | 93.86 | 85.21 | 78.87 |
| TS13 | 22,774,339 | 44.88 | 94.37 | 88.07 | 81.76 |
| TS21 | 22,879,808 | 44.84 | 94.52 | 88.81 | 82.58 |
| TS22 | 21,572,083 | 44.99 | 94.35 | 89.24 | 82.54 |
| TS23 | 21,401,733 | 44.76 | 94.50 | 88.25 | 82.18 |

# **Supplementary** **Figure 1** Rooting type of tea cuttings. A, callus rooting type; B, cortex rooting type.


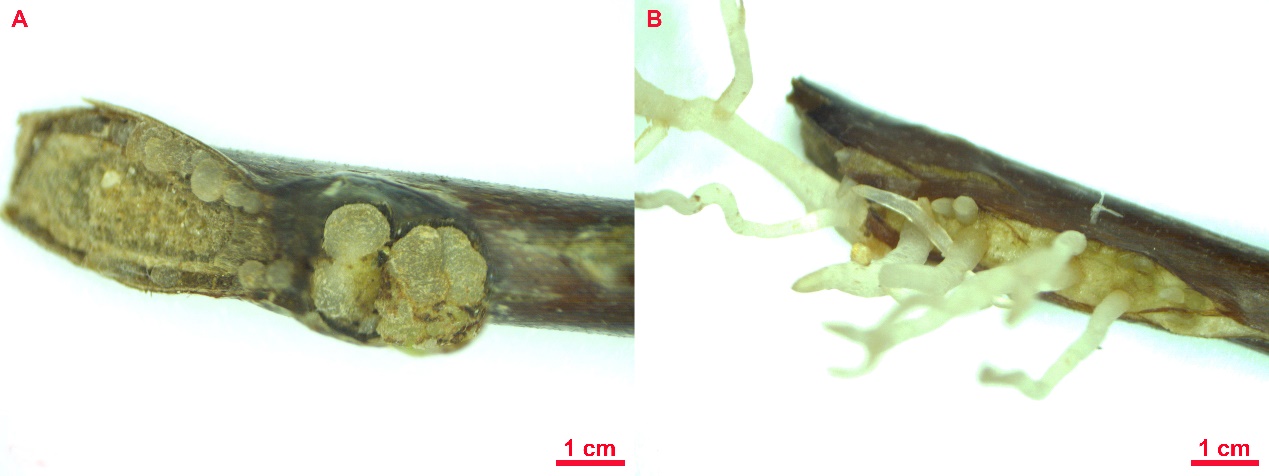


# **Supplementary Figure 2** Status of mycorrhizal colonization in clover roots. C and T indicated non-mycorrhizal and mycorrhizal clover root, respectively; Red arrow pointed to the vesicle of AMF.


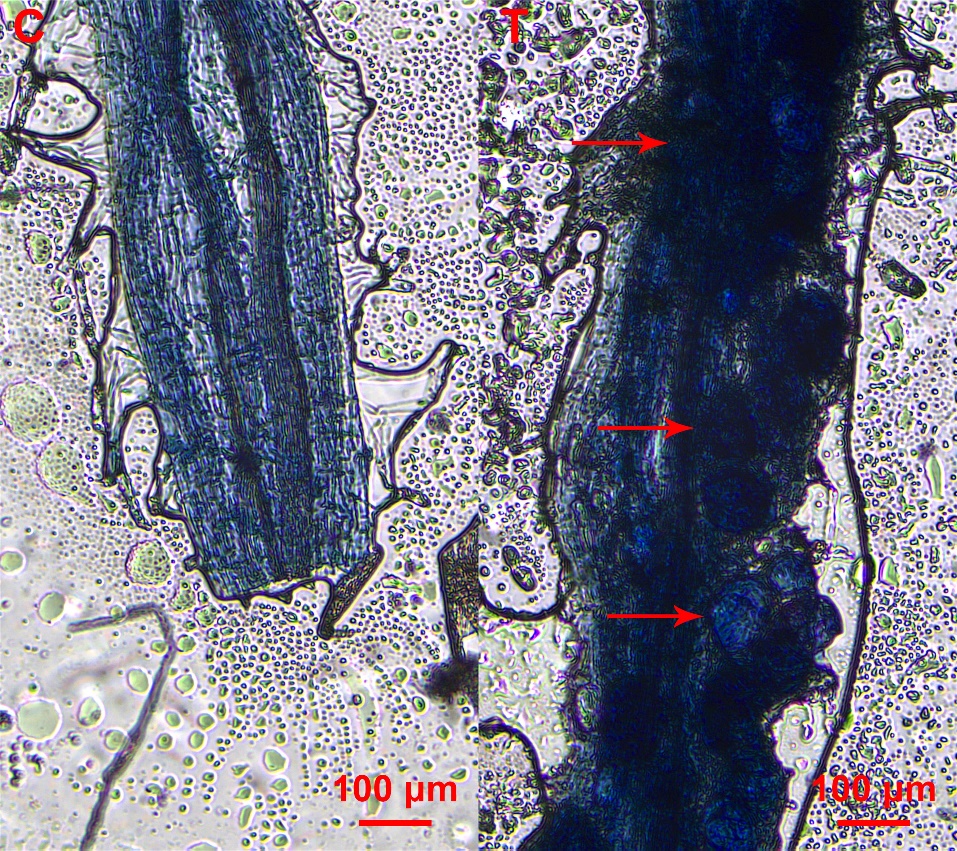


# **Supplementary Figure 3** Effects of different treatments on rooting of cuttings in various tea plant varieties. C, non-mycorrhizal treatment; T, mycorrhizal treatment; Figure A, ‘Pingyangtezao’; Figure B, ‘Longjing 43’; Figure C, ‘Longjingchangye’; S0, S1, S2 and S3 indicated stages of non-rooting, AR protrusion, AR formation and AR elongation, respectively.


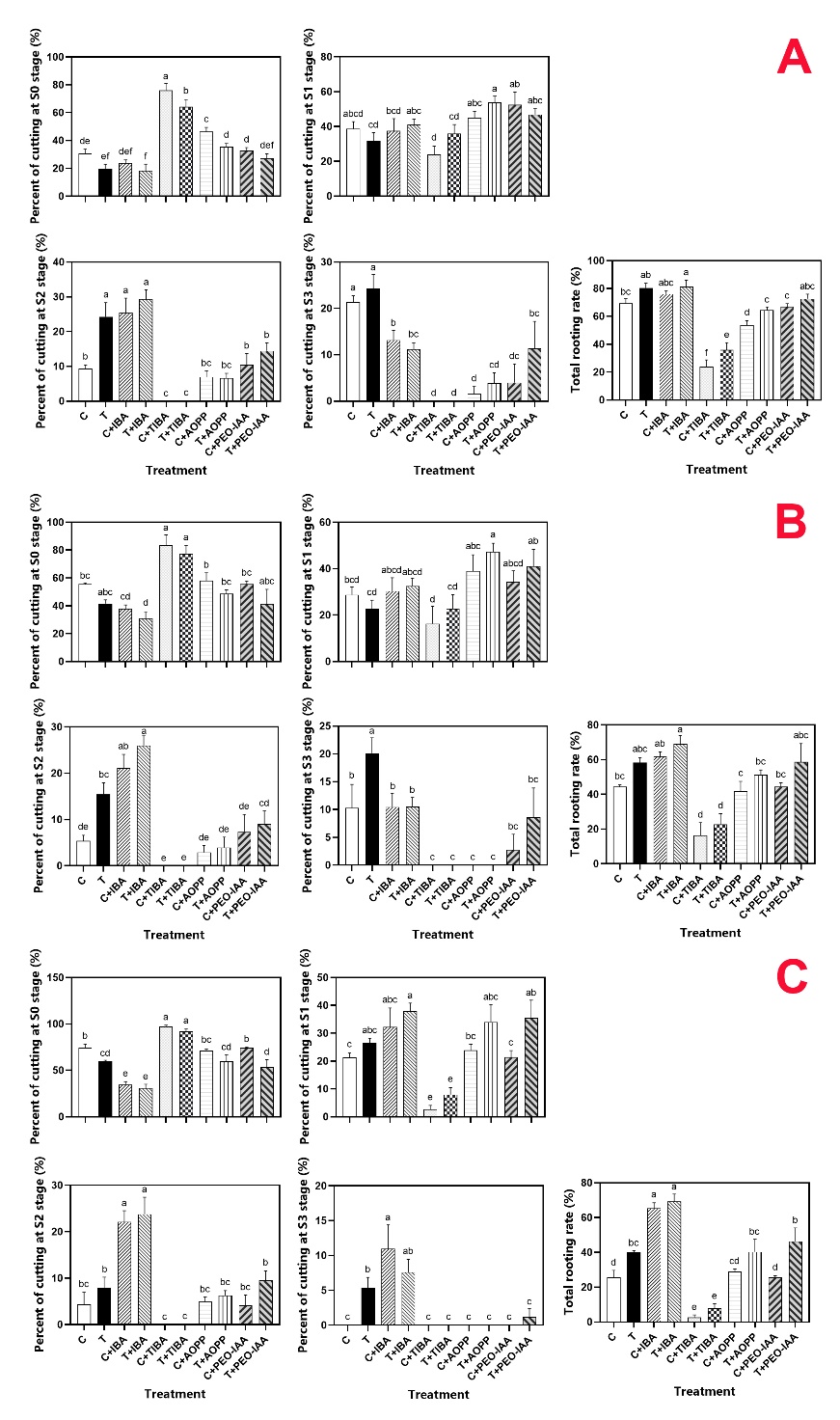


# **Supplementary Figure 4** Validation of RNA-seq results by qRT-PCR. Data from qRT-PCR (Black-filled circle) and RNA-Seq (White column) of 11 selected genes were means of three replicates and bars represent Standard Error. C, non-mycorrhizal treatment; T, mycorrhizal treatment; S0, S1, and S2 indicated stages of non-rooting, AR protrusion, and AR formation, respectively.


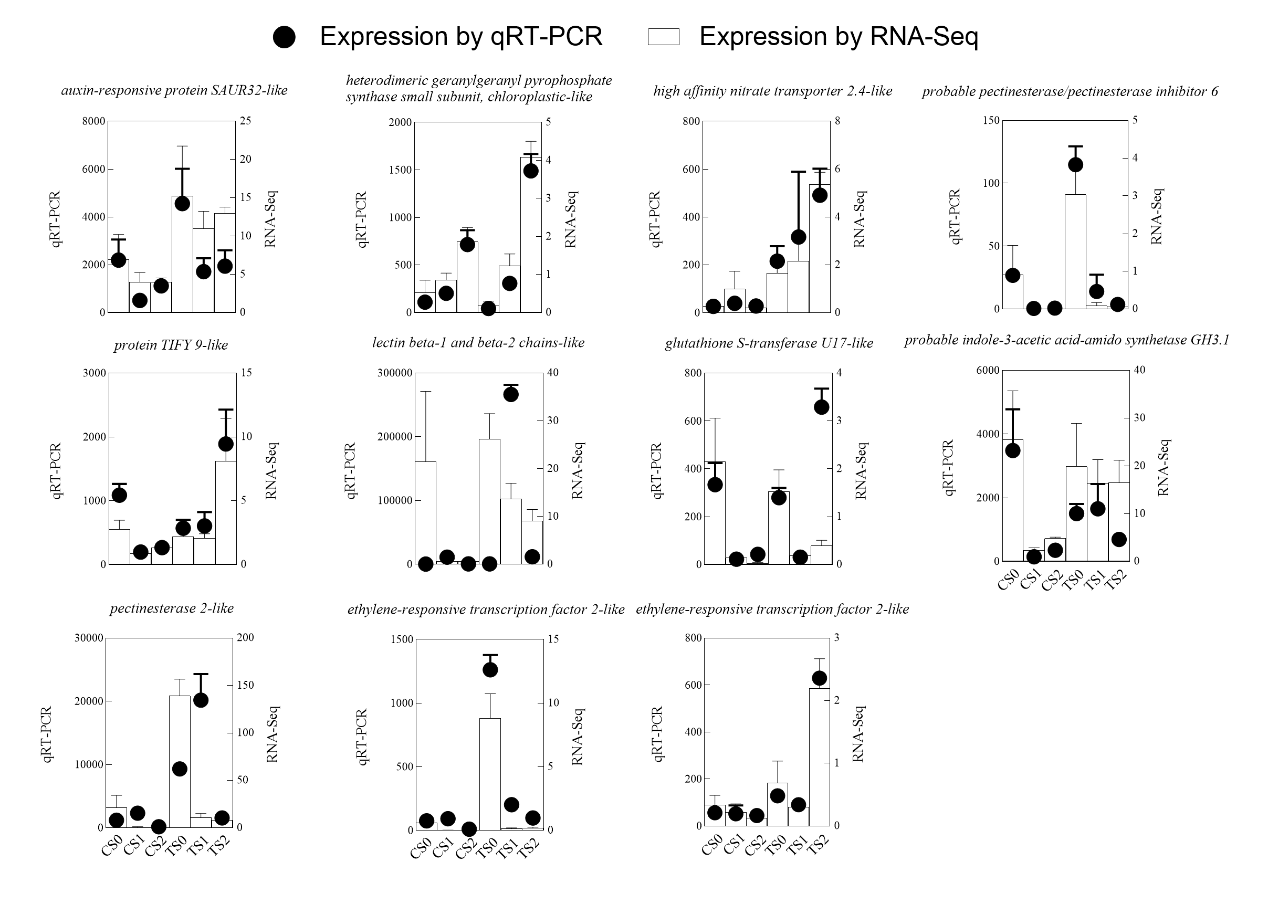


# **Supplementary Figure 5** Correlation analysis of fold change data between qRT-PCR and RNA-seq. Scatterplots were generated by the log2(Fold change) from RNA-seq (x-axis) and qRT-PCR (y-axis).


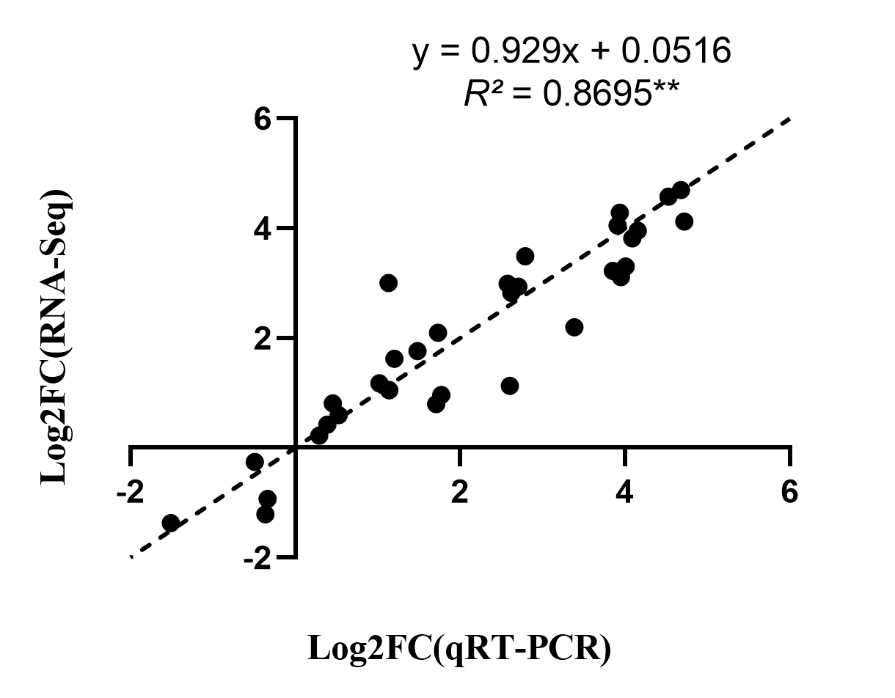

Supplement: Supplementary file 1 [file DataSheet_1.zip › Supplementary Tables and Figures.DOCX]
